# Night-time lights: A global, long term look at links to socio-economic trends

**DOI:** 10.1371/journal.pone.0174610

**Published:** 2017-03-27

**Authors:** Jeremy Proville, Daniel Zavala-Araiza, Gernot Wagner

**Affiliations:** 1 Office of Economic Policy and Analysis, Environmental Defense Fund, New York, New York, United States of America; 2 Climate and Energy Program, Environmental Defense Fund, Austin, Texas, United States of America; 3 John A. Paulson School of Engineering and Applied Sciences, Harvard University, and Harvard University Center for the Environment, Cambridge, Massachusetts, United States of America; Bristol University/Remote Sensing Solutions Inc., UNITED STATES

## Abstract

We use a parallelized spatial analytics platform to process the twenty-one year totality of the longest-running time series of night-time lights data—the Defense Meteorological Satellite Program (DMSP) dataset—surpassing the narrower scope of prior studies to assess changes in area lit of countries globally. Doing so allows a retrospective look at the global, long-term relationships between night-time lights and a series of socio-economic indicators. We find the strongest correlations with electricity consumption, CO_2_ emissions, and GDP, followed by population, CH_4_ emissions, N_2_O emissions, poverty (inverse) and F-gas emissions. Relating area lit to electricity consumption shows that while a basic linear model provides a good statistical fit, regional and temporal trends are found to have a significant impact.

## Introduction

Human activities have transformed over half of the global land surface [1], a trend that continues to increase and is apparent in satellite imagery. One of the clearest signs is night-time lights as seen from space. Two central datasets are those derived from the Defense Meteorological Satellite Program (DMSP) and its successor, the Visible Infrared Imaging Radiometer Suite (VIIRS). There is a long literature exploring the imagery provided by these products, and the wide variety of applications they can serve. Perhaps most importantly, they are able to inform our understanding about the relationship between human activities and our environment at a global scale, without relying on national statistics with oft-differing methodologies and motivations by those collecting them.

DMSP data are the longest-running time series of night-time lights, dating back to 1992 [2]. Over this period, a great deal of topics has been explored, at various spatial scales. At finer geographical scales, for example, Mellander et al. [3] have had success in using DMSP as a proxy for certain indicators in Sweden (e.g. population, establishment density); many similar analyses have been done for other regions [4,5,6,7,8,9]. At larger scales, DMSP has been used for everything from generating detailed CO_2_ emission maps [10,11] to creating innovative development indices [12] to estimating natural gas flaring trends [13], among many others [14,15,16].

Several such global studies have explored the basic links and correlations between DMSP data and other well-documented variables, such as population [17], CO_2_ [18], GDP and electric power consumption [19]. These relationships provide insight into the value of using night-time lights as descriptors and proxies for human activity—both economic and environmental. One impediment to obtaining a better understanding of such relationships has been the computational limitations of dealing with these datasets, which consist of a large catalog of sizeable images. As such, most of the analyses exploring broad, national correlations have had to narrow their focus either in terms of temporal or spatial scales. For example, Doll et al. [18] and Elvidge et al. [19] constrained their analysis to a composite of DMSP observations over a six-month period.

We use Google Earth Engine (GEE), a platform recently made available to researchers that allows users to overcome some of the computational limitations of earlier efforts, to explore more comprehensive global aggregate relationships at national scales between DMSP and a series of economic and environmental variables. While GEE itself is still under development, it has already provided great value to the research community: from deriving high resolution datasets on global forest change [20], to settlement mapping [21,22,23]. Many other emerging cloud computing providers and frameworks currently exist and excel in these types of analyses, such as Hadoop and Spark.

The following sections describe our methods and results in summarizing GEE data for 246 nation-states, across a twenty-one year record (1992–2013). Both the data used and our methods are freely available for further exploration by others wishing to employ night-time lights for broader study.

## Methods

### Input datasets

Our night-time lights input dataset consists of annual composites of the stable lights band from DMSP-OLS Nighttime Lights Time Series Version 4, spanning 1992–2013 [2]. In years with two annual composites, we use data from newer satellites. For the year 2002, data have not been composited north of a latitude of ~58°N—impacted regions are omitted from the final dataset for that single year (see S1 Table).

We use the Thematic Mapping World Borders Dataset [24] for administrative boundaries of countries and nation-states. The use of this, rather than a more narrow definition for national boundaries (such as United Nations members) accomplishes two goals: it allows us to further disaggregate our analysis, and provides greater flexibility for users of our resulting dataset to re-allocate and define territories according to their needs. Only a small subset of this dataset is composed of nation-states. For simplicity, we include these in our definition of countries throughout.

Data on Gross Domestic Product (GDP; nominal, current US$ levels), poverty headcount ratio (at national poverty lines) and population are from the World Bank [25]. Population estimates are composed from a combination of United Nations, Eurostat and national census data. Electric power consumption (in billion kWh) is taken from the Energy Information Administration [26]. CO_2_ emission estimates are obtained from CDIAC [27], while other greenhouse-gas data are taken from EDGAR [28]. We have eliminated extremely low emission values for F-gases (less than 50 kg per year) from the analysis. This eliminates a slight bimodal peak in the resulting logarithmic distribution. All of the input datasets listed above are freely available without restriction. With the exception of electricity consumption data, all indicators can be downloaded via the World Bank data portal [25] (also see S2 Table).

### Metric selection

Various metrics have been proposed and used for exposing relationships between night-time lights and other variables. Among them are “sum of lights” (aggregating intensity values) [11,29], developing a “lights index” (Elvidge, et al., 2009), or even comparing statistics across unique digital number (DN) values from the DMSP images [17]. We have used an “area lit” metric, with a threshold value applied to each grid cell. This approach is similar to Elvidge et al. [31,19] and Doll et al. [18] but differs in the threshold chosen. We use a DN value of 31; values in this band range up to 63. This represents a balanced selection aimed towards ruling out pixels of smaller, potentially temporary or interannual lighting while also capturing the vast majority of persistently lit areas. Trend analysis using alternative DN values indicates that results are not particularly sensitive to the specific threshold chosen. Nonetheless, threshold selection has shown itself to be an important consideration [32]. This simple thresholding approach can be mapped as a server-side algorithm in GEE, and distributed as independent parallel tests across the array of raster values. The pixel counts are subsequently converted into their equivalent areal coverages in square km. See S1 File for the code used and a link to the associated GEE workspace. This particular metric and analytical platform provided an efficient means of extracting results; however, other combinations may yield greater computational efficiency.

Despite a great deal of pre-processing and corrections performed for DMSP Version 4 images, there remains a known saturation effect at higher levels in the stable lights band [33,34]. A known approach to algorithmically correcting for this as postulated by Letu et al. [33] is oriented towards regional/city level analyses. For subsets of the imagery where lunar illumination (and the DMSP sensor’s gain setting) are low, NOAA provides calibrated data [2]. Nonetheless, the optimization and selection of an un-adjusted threshold value from the stable lights band, as we have done, performs well at aggregated scales. In part, this is due to use of a binary assignment for pixel values (lit or not) in combination with a threshold low enough to not be adversely biased by the saturation effect.

### GIS framework and statistical analysis

The final dataset used for our statistical analyses represents an estimate of lit area (in square km) by country, summarized at a 0.5x0.5 km grid cell resolution. Aside from poverty headcount ratio, we use logarithmic variables to accommodate large variations. The code, using R, and data file (a pre-formatted equivalent of S2 Table) can be found in S2 and S3 Files, respectively.

## Results

### Simple correlations

**Fig 1:**
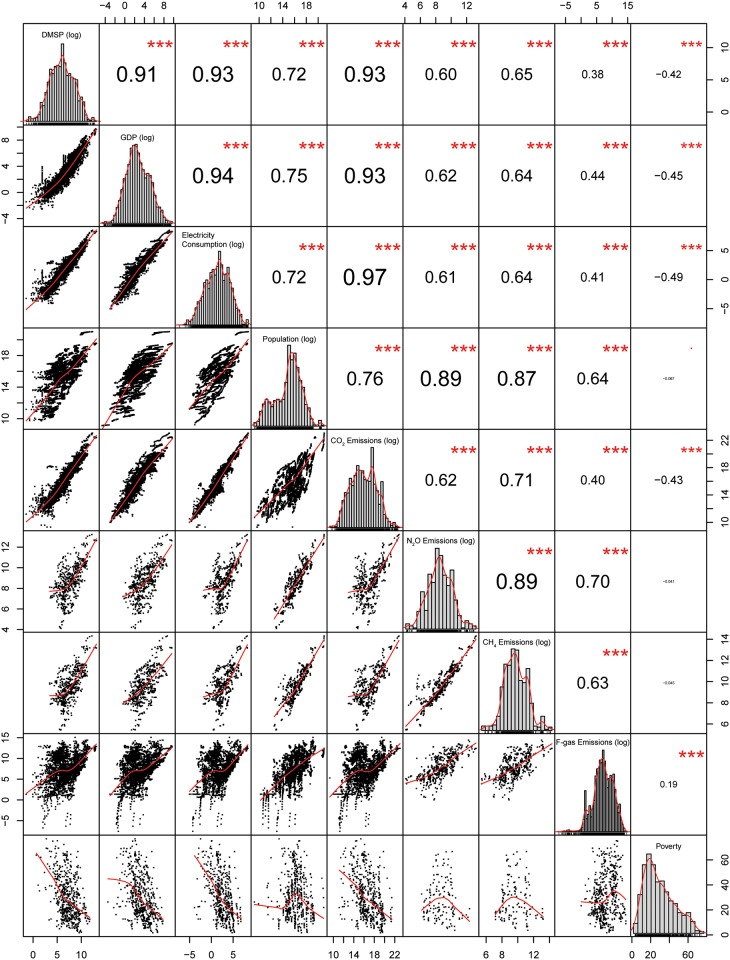
Correlation between area lit and a collection of socio-economic indicators. The matrix above shows links between logarithms of Area Lit, GDP, Electric Power Consumption, Population, CO_2_ Emissions, N_2_O Emissions, CH_4_ Emissions, F-gas Emissions, and non-log Poverty Headcount Ratio, respectively. Numbers on the top-right side of the matrix denote Pearson’s ***r*** values (font size ***∝*** value), and stars represent significance level (***, ***p* < 0.05**).

Fig 1 illustrates correlations between the area lit from night-time lights, and a series of economic and environmental variables (see S2 Table for data). The number of observations across most pairs was high given the long data record (3444 < *n* < 4269), except for N_2_O, CH_4_ and poverty headcount ratio data, where inventories or data years were less frequent (536 < *n* < 593). Area lit correlates highly with electric power consumption, GDP, and CO_2_ emissions (0.91 < *r* < 0.93). Non-CO_2_ greenhouse gases correlate less directly (0.38 < *r* < 0.65), as would be expected due to the fact that they are by-products of activities further removed from fossil fuel burning and electricity generation. Many of these sources (such as agriculture and industrial processes) are not readily perceived through night-time illumination. A metric of poverty headcount ratio, standardized at national levels, correlated negatively (*r* = -0.42). Adjusting for the total area of countries (by using a logarithm of ‘percent area lit’, rather than an absolute measure in square km) provides a stronger correlation (*r* = -0.57). These findings support the notion that countries with higher poverty rates exhibit relatively less night-time illumination than their counterparts. Fig 1 presents a full matrix to highlight the fact that there is a high degree of correlation between many of the non-DMSP variables themselves.

An accompanying motion chart relating all of the datasets listed in Fig 1 is available online [35]. This visualization allows users to observe how the correlations between any combination of variables evolve over the 21-year data record. It also enables a multivariate interpretation of results, by allowing data to be assigned to the color and size of markers on the chart.

R^2^ values for GDP and CO_2_ emissions were consistent with the findings in Doll et al. [18]. Our correlations for GDP, electric power consumption, and population, however, are lower compared to Elvidge et al.’s [31] smaller sample of 21 countries over the period 1994/1995. While a subsequent analysis [19] (expanding their sample to 200 countries for the same period) does not report goodness-of-fit values, overall trends mirror ours. Result suggest that these close relationships hold over the longer term.

Our analysis reflects a larger pool of countries and years—in turn increasing variability in the dataset, stemming from an expanded set of economic conditions and forms of governance. Compositional analysis in the context of villages in Vietnam, has shown that DMSP intensity values are typically driven in large part by electrified homes and streetlights [36]. Similarly to Doll et al. [18], we find that centrally-planned economies (notably North Korea, China and Russia) tend to be outliers, further supporting their hypothesis that these countries have lower levels of residential and/or street lighting than equally developed counterparts. Further, we find supporting evidence for Elvidge et al.’s [19] finding that more economically prosperous nations exhibit anomalously high levels of lit area relative to their population, and vice versa for poorer countries.

### Regression analysis

Fig 1 shows that electric power consumption, CO_2_ emissions, GDP, and population exhibit the strongest correlations with area lit. It is also important to note that these parameters are correlated amongst themselves, and thus lead to collinearity in the context of a multivariate linear regression model. We first explore single paired relationships. The basic model specification follows the form:

$$\text{ln}\left(DMSP\right)=\alpha +\beta_{o}+\;\beta_{1}x+\varepsilon$$ (1)

where ln(*DMSP*) is the logarithm of area lit (in sq. km), *α* is the intercept, *β*_1_ is a coefficient for the independent variable *x*, and *ε* is the residual standard error of the model. *β*_0_ encapsulates fixed effects, according to:

$$\beta_{o}=\beta_{region}x_{region}+\beta_{year}x_{year}+\beta_{country}x_{country}$$ (2)

Table 1 demonstrates how fixing effects alternately on regions, countries and years affects goodness-of-fit and standard error.

**Table 1:** Comparison of regression models between DMSP (logarithm) and electricity consumption (logarithm). Describes regression outputs when fixing effects for various dimensions in the data, both individually and in combination.

**Fixed Effects:**	**None**	**Regions**	**Countries**
***β*_1_**	0.907 ***	0.965 ***	0.803 ***
	(0.0056)	(0.0062)	(0.0175)
***α***	4.89 ***	-	-
***β*_*region*_**	-	[4.18 to 5.18] ***	-
***β*_*country*_**	-	-	[1.35 to 7.03] ***
***ε***	0.878	0.826	0.393
**R^2^_A_**	0.864	0.986	0.997
**Fixed Effects:**	**Years**	**Regions & Years**	**Countries & Years**
***β*_1_**	0.908 ***	0.966 ***	0.466 ***
	(0.0055)	(0.0061)	(0.0227)
***β*_*region*_**	-	[-1.01 to -0.158] ***	-
***β*_*country*_**	-	-	[-3.79 to 4.14] ***
***β*_*year*_**	[4.59 to 5.24] ***	[4.86 to 5.18] ***	[4.59 to 5.43] ***
***ε***	0.864	0.811	0.333
**R^2^_A_**	0.984	0.986	0.998

Signif. codes:

‘***’ 0.001. *n* = 4,197

**Comparison of Regression Models for** *x* = ln(*Electricity*)

A basic linear model relating the logarithms of area lit to electricity consumption alone provides a good fit, confirming simple correlation analyses above. Accounting for fixed effects from various spatial scales (regions, countries), years, and combinations of both further improves fit.

**Fig 2:**
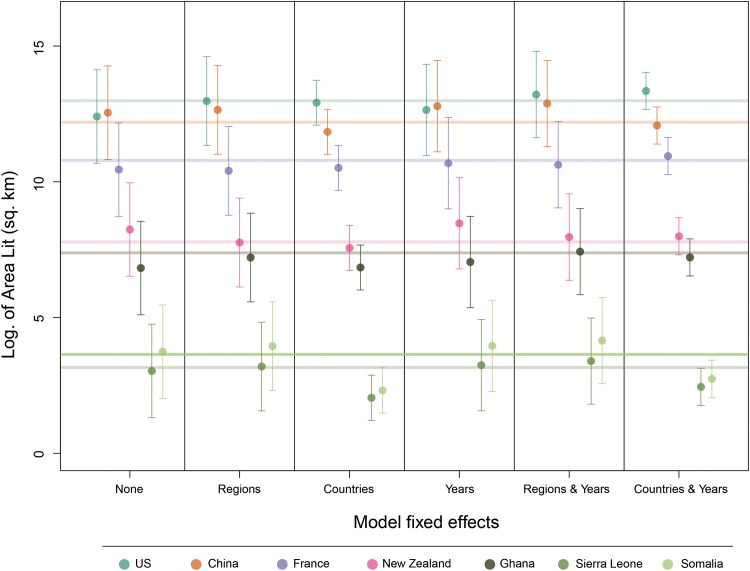
Comparison of predicted area lit values as a function of energy consumption, for different countries for the year 2012. We selected 7 countries from different regions and use the mean logarithm of energy consumption for each country for 2012 as the input to the six models described in Table 1. Horizontal bars represent the observed area lit values, while error bars depict a 95% confidence interval.

Fig 2 provides a visual depiction of how predictions in area lit differ amongst models presented in Table 1, in contrast to observed values. For the sake of providing an illustrative comparison, we arbitrarily selected the year 2012 and 7 countries from different regions: US, China, France, New Zealand, Ghana, Sierra Leone and Somalia. Note the location of the points (predicted) relative to the horizontal lines (observed).

Moving from a model without fixed effects to the ones that incorporate spatial and temporal information reduces the relative discrepancy between observed and predicted values. In a general sense, all models provide a statistically significant prediction of the area lit as a function of electricity consumption. Nonetheless, incorporating information of the specific country of interest reduces the size of the error term roughly by half. While the representative regression analysis pertains to electricity use, the relative importance of spatial and temporal heterogeneity is quite similar for other socio-economic indicators, notably CO_2_ emissions and GDP.

## Discussion

Few prior studies have explored long-term temporal trends over large areas using the DMSP annual composites. The most notable study is Bennie et al. [37], an in-depth analysis of changes in brightness in Europe between the years 1995–2000 and 2005–2010. Their respective method differs from ours by assessing changes in DN values, rather than thresholding. We reach the same conclusions regarding the overall trend in the raw data, while our methodology allows for a continuous evaluation from 1992 to 2013.

We perform a sensitivity analysis to understand how the omission of specific years or regions affects model fit. Adjusting data years does not have a large impact on goodness-of-fit, yet certain combinations of regions and indicators do. For GDP, electricity consumption, and CO_2_ emissions, omission of the Americas decreases fit (r^2^ ↓ 0.02, 0.03, 0.02, respectively), while for Asia fit increases (r^2^ ↑ 0.04, 0.03, 0.02). In the case of population, fit is greatly improved when omitting Africa (r^2^ ↑ 0.15), and impaired when omitting the Americas (r^2^ ↓ 0.07). These findings seem intuitive given the prevalence of countries with better statistical reporting in the Americas, and vice versa with developing countries in Asia and Africa. Chen and Nordhaus [38] document this effect, and we also find it to be demonstrated in comparing certain countries within Fig 2. Ghana, in our example, is relatively wealthy and bears a more reliable degree of statistical reporting than other African nations, such as Sierra Leone or Somalia. Plotting the latter countries produces a mean predicted area lit lower than observed levels; this result is anticipated given that what we expect using reported data does not match what is observed from satellite records.

One key area of focus for future improvements to our method would be to find ways to implement calibrations proposed by other researchers [13,30,33,39] on DMSP imagery in the analytical framework outlined above. Within the GEE platform, we expect this to become feasible in the future as the product continues to develop, and as new datasets are added. While we do not believe the lack of calibration, according to the methods cited above, would greatly affect our findings (given that we have chosen to use an area lit thresholding approach), this would improve the accuracy of the estimates. It should be noted that fully calibrating and removing sources of variation across years is ultimately very challenging. One such factor is that a total of six satellites were collecting imagery over the data record, each with differently calibrated sensors. Extemporaneous adjustments to instrument gain that were made during orbit further complicates calibration [37].

From a methodological standpoint, prior studies using the DMSP dataset rarely provide a detailed description of the GIS software used and computational approach employed in deriving spatial statistics. Ours is performed in a distributed environment, and illustrates a case where a simple operation (i.e. counting pixels above a certain threshold within polygons) is being processed in parallel across a large raster image catalog. As the successor to DMSP for night-time light sensing, imagery from the VIIRS mission is clearly superior [40]. Yet, the data record is still relatively short: standardized, reliable data begin in January 2014. The increased resolution of VIIRS presents great promise for better understanding relationships between night-time lights and human activity. For example, Ou et al. [41] have used VIIRS imagery in mapping fine-grained spatial distributions of CO_2_ emissions in Chinese cities, while Shi et al. [42] have done so for GDP and electric power consumption. Further examples are rapidly emerging [43,44,45,46].

One of the more typical uses of night-time lighting imagery is to serve as a proxy measure for other indicators. Assessing economic activity is perhaps the most prevalent application, as pioneered by Doll et al. [4]. Yet, it is important to consider the limitations of such approaches. Night-time lights are unlikely to provide added value as a proxy in countries with good statistical systems, due to the high measurement error as compared to national inventories [38,47]. Sutton et al. [48] agree, though conclude that night-time lights still provide useful insights into estimating informal economic activity; Ghosh et al. [49] go one step further, assessing the informal sector in an empirical case study of Mexico.

The work of Shi et al. [44] and Jean et al. [50] provide excellent recent examples of the value obtained when combining these proxy approaches with the increased resolution of VIIRS and with machine learning, respectively. These studies highlight the fact that future research is rife with opportunities to learn more about our world by marrying large datasets with powerful computational tools.

## Conclusions

Over the course of a twenty-year data record and at aggregated scales, we find high correlations between the area lit from night-time lights on the one hand, and GDP, electricity consumption, and CO_2_ emissions on the other. Correlations with population, N_2_O, and CH_4_ emissions are still slightly less high, while we find moderate correlations with F-gas emissions and an inverse measure of poverty. To this end, our findings are largely consistent with prior studies having a narrower geographical or temporal focus.

Variability in night-time lights can be explained in large part by electricity use in a basic logarithmic regression model. A comparison of alternative fixed effects specifications underscores significant temporal and spatial aspects to the data. Controlling for heterogeneity across regions and years increases goodness-of-fit, likely explained by differences in governance and harmonized global economic cycles, respectively.

Platforms such as GEE that provide the means for distributed parallel processing help overcome some of the computational challenges inherent in such large datasets. We hope our application demonstrates the value of such platforms for GIS researchers and those relying on their output.

## Supporting information

S1 File Code for Google Earth Engine used to extract measures of lit area. This file contains the Javascript code used in the Google Earth Engine platform, in order to derive estimates of lit areas by country. The header of this file contains a URL to the associated workspace.
(JS) Click here for additional data file.

S2 File Code for statistical analysis. This file contains the R code used in preparing statistical estimates for Figs 1 and 2 and Table 1 of the main text.
(R) Click here for additional data file.

S3 File Data file for use with statistical analysis. This is a comma-separated values (csv) file containing the raw data for use with the R code in S2 File.
(CSV)

S1 Table Regions omitted from the final dataset for the year 2002. These regions were omitted due to lack of data north of ~58°N, for that specific annual composite image.
(XLSX)

S2 Table Full dataset. This is a spreadsheet containing all data used in our statistical analysis, containing area lit and all other socio-economic indicators, broken down by nation-state and year for the period 1992–2013.
(XLSX)
